# Learning and teaching sustainable business in the digital era: a connectivism theory approach

**DOI:** 10.1186/s41239-023-00390-w

**Published:** 2023-04-19

**Authors:** Olga Dziubaniuk, Maria Ivanova-Gongne, Monica Nyholm

**Affiliations:** 1 School of Business and Economics, Åbo Akademi University, Vänrikinkatu 3B, 20500 Turku, Finland; 2 Unit of Industrial Engineering and Management, Tampere University, Korkeakoulunkatu 7, 33720 Tampere, Finland

**Keywords:** Connectivism theory, Higher education institution, Learning environment, Digital technologies, Sustainable development, Sustainable business

## Abstract

Higher education institutions may adopt various approaches to the pedagogic principles and methods used in teaching sustainable development in business and marketing courses. These methods can include the utilisation of digital technologies and online communication to facilitate distance learning and fast access to relevant information. Changes towards the digitalisation of the learning environment especially gained popularity during the Covid-19 pandemic. In the post-pandemic period, digitalisation continues to facilitate the learning and teaching processes. However, the implementation of digital technologies, besides technological expertise, requires appropriate theoretical frameworks for understanding how learning is developed. This study explores connectivism theory applied to the pedagogic practices of knowledge dissemination concerning sustainable development in the fields of business and marketing. Connectivism embraces knowledge as a network where the learner, with the help of digital technologies, develops mental connections between pieces of information during interaction with various information sources. This qualitative research empirically explores the principles of connectivism embedded in the learning and teaching of a university course conducted online. The research findings indicate that connectivism may be a suitable conceptual framework that motivates learners to develop knowledge through digital enablers, discussions and social networking and to make connections to sustainability concepts. The principles of connectivism may help instructors to develop a learning environment where learners add understandings to their previous knowledge on sustainability through online interactions and by accessing digital knowledge sources. This study makes several interdisciplinary contributions by deepening the insights into digital pedagogic methods and approaches for the facilitation of learning, which may be of interest to academic and other pedagogic practitioners.

## Introduction

Technology has become an inevitable part of the learning process at higher education institutions (HEIs). The recent Covid-19 pandemic especially increased the utilisation of digital technologies by making universities switch to online or hybrid modes of teaching and learning (Al-Mutairi & Mubayrik, 2021; Pokhrel & Chhetri, 2021). Simultaneously, pedagogic advancement occurs in international business studies (Aggarwal & Wu, 2020; Kardes, 2020). This especially concerns courses dedicated to sustainable development (SD) in business (Bagur-Femenías et al., 2020; Montiel et al., 2020). The learning of sustainability in business is frequently grounded on theories, such as constructivism (Dziubaniuk & Nyholm, 2020), social learning theory (Keen et al., 2005), transformative learning (Seatter & Ceulemans, 2017) and other pedagogical frameworks, such as experiential learning (Anastasiadis, 2020), active learning (Claro & Esteves, 2021) and design thinking (Manna et al., 2022). However, the integration of digital technologies into the learning process may require a revision of the conventional learning theories applied to curriculum design within business studies. The digital age demands new approaches to the facilitation of students’ learning, including new ontological and epistemological approaches to organising the learning environment with the help of information and communication technologies.

The utilisation of digital technologies for organising the learning environment has become ‘a new normal’ in universities and may even be more used in the future to bridge online and offline learning. As online learning continues to be further integrated into the foundations of higher education, digital teaching skills and student support must be a priority in the pedagogy of online teaching (Kordrostami & Seitz, 2022; Simamora, 2020). New approaches to facilitating digital learning also concern courses in international business and marketing, with a focus on sustainable development. The theory of connectivism can be useful for the design of the learning environment. *Connectivism learning theory* can be a suitable alternative in cases where students develop knowledge by forming social networks with the help of technology (AlDahdouh, 2018; Siemens, 2005). Connectivism is a theoretical framework for the understanding of learning, where learners “*make connections between ideas located throughout their personal learning networks, which are composed of numerous information resources and technologies*” (Dunaway, 2011, p. 676). Thus, connectivism helps identify the enablers of learning, stemming from technological advancements and the design of an appropriate learning environment.

Connectivism is a relatively new learning theory (Kop & Hill, 2008) that has found its application in organisational leadership studies (Corbett & Spinello, 2020), medical studies (Goldie, 2016), intercultural management (Shrivastava, 2018) and virtual teaching in educational studies (Barnett et al., 2013). Although connectivism has received wide attention, unlike other learning theories, it lacks a lengthy history of testing and revision to arrive at a definitive framework for understanding how people learn differently in the digital world (Corbett & Spinello, 2020). Furthermore, few studies have addressed connectivism in relation to the learning of sustainability in business studies (Abad-Segura et al., 2020; Karlusch et al., 2018), whereas digital technologies are commonly applied in teaching sustainability at universities (Rof et al., 2020).

Concepts of SD are embedded in a wide variety of subjects within an HEI, and the importance of learning about them is stressed, promoted and supported by national and international organisations (Mulà & Tilbury, 2009; UNESCO, 2022). Knowledge of SD is demanded by employers in modern labour markets (Zawacki‐Richter, 2021), as is the ability of learners to continue learning after graduating from HEIs and to grasp constant changes related to sustainability. SD possesses a specific place in business studies, where the aim is to educate business and social leaders (Adams et al., 2011; Jónsson et al., 2021; Sandri, 2022). To increase students’ knowledge of SD, educators need to challenge existing teaching methodologies and rethink organisational learning processes to fit modern realities (Filho et al., 2018).

Currently, SD and business studies are often conducted online or in a hybrid mode that emphasises the role of technologies in the learning process but raises questions about the efficiency of learning. Although digital technologies as enablers of learning in SD-related subjects have received the attention of some researchers (e.g. Bagur-Femenías et al., 2020; Bush et al., 2016; Rashid, 2019), the conceptual approach to the framing of the digital learning environment has still been rarely investigated in this context. Connectivism can be a promising framework for learning in sustainability-related courses, where students can make mental connections between SD concepts and become motivated to keep searching for relevant information (Gómez-Zermeño, 2020; Wals, 2011). Connectivism can help in structurally approaching large amounts of information on sustainability regulations and trend changes; in grasping the interplay between the social, environmental and economic domains of SD; and in individualising learning (e.g. Gallagher, 2018; Karlusch et al., 2018; Utecht & Keller, 2019; Klašnja-Milićević & Ivanović, 2021).

This study aims *to explore how teaching and learning sustainability in business can be approached in a digital learning environment through the principles of connectivism.* To address the study objectives, connectivism theory to qualitative research based on empirical data collected in the form of open-ended written course feedback by students who attended the Sustainable Business course at Åbo Akademi University (ÅAU), Finland, in 2020 and 2021. During these two years, the pedagogic mode of this course had to be switched from in-class to online teaching due to the Covid-19 pandemic. ÅAU actively attempts to address sustainability issues in teaching and research, as stated in the university strategy (Åbo Akademi, 2020). In general, Finland has a favourable educational environment for integrating sustainability into university-level studies because of the country’s education policy supporting SD and high public awareness of sustainability challenges (Friman et al., 2018; Jónsson et al., 2021; Ministry of Education & Culture, 2020).

This study makes several interdisciplinary contributions. Primarily, it contributes to the literature on education for sustainability (e.g. Kopnina, 2020), placing an emphasis on bridging business studies and principles of SD. This research adds to the literature on students’ learning by suggesting and exploring connectivism theory for learning sustainable business in an online environment, which has rarely been addressed by pedagogy scholars (Abad-Segura et al., 2020; Davidson et al., 2021; Gómez-Zermeño, 2020). The empirical and conceptual contributions of this study aim to extend knowledge on connectivism theory, which is often criticised for lacking boundaries and deeper conceptualisation (Oommen, 2020). The study illustrates a potential development of the teaching methodology towards further utilisation of digital technologies, which, besides being convenient in organising the study process, also affects the students’ perception of information (Bond et al., 2018; Waycott et al., 2010). Despite the empirical focus on the Finnish educational context, this study may be of interest to educators in HEIs in other countries and other educators facilitating learning and teaching with digital technologies. Finally, this study contributes to the development of educational practices for sustainability, as identified in the UN Sustainable Development Goal 4 ‘Quality Education’ (SDG, 2015).

This article is structured as follows. The next section presents a conceptual overview of teaching and learning in the scope of higher education for SD, followed by a literature review on connectivism theory and digital learning. The Methodology section describes the approaches to the collection and interpretation of empirical data. The Results and Discussion sections illustrate how the theory of connectivism is interconnected with students’ learning outcomes and their processes of knowledge development. The Conclusion section summarises the research findings, contributions and study limitations and proposes several future research avenues.

## Literature review

### Teaching and learning sustainability in business

SD is among the core topics on the global agenda being labelled ‘a long-term goal (i.e. a more sustainable world)’ and is related to ‘the processes and pathways to achieve it’ (UNESCO, 2022). In 2015, the United Nations introduced 17 SD Goals (SDGs), which concern all areas of human activity and require individuals, organisations and institutions to take action in order to achieve these goals (SDG, 2015). HEIs are instrumental in this process and should incorporate SDGs in teaching within all disciplines and facilitate knowledge creation about sustainability, which would allow the students to apply sustainability principles in their future professions and life in general (Merritt et al., 2018; Mulà & Tilbury, 2009). The implementation of sustainability in HEIs should be conducted throughout various layers (Lozano & Young, 2013), with education being one of those most studied in research (Lozano et al., 2015).

Business schools have a special role in reaching SDGs, considering that they educate future leaders of for-profit and non-profit organisations, which are important actors in implementing sustainability within society (Dziubaniuk & Nyholm, 2020; Sandri, 2022). In an already classic paper, Sterling (2004) outlined three ways of teaching sustainability: (1) educating *about* sustainability, which involves adding sustainability modules to the educational offering; (2) education *for* sustainability, which focuses on the transformation of the entire institution by adopting sustainable approaches; and (3) *capacity building*, which focuses on transforming students by developing their skills for sustainability (Painter-Morland et al., 2016). Business schools require a more transformative approach to sustainability education, with the development of skills for sustainability as the main focus (ibid.). According to several studies, the core challenges of teaching sustainability in business schools are the following (Adams et al., 2011; Bien & Sassen, 2020; Filho, 2020; Janoušková et al., 2019):

1. Facilitating a shift in thinking from a seller–customer view to a more holistic stakeholder approach by considering how a company adds value to a broad range of stakeholders.
2. Improving critical self-reflection skills, which will enable proper reflection on managerial actions and decision-making processes.
3. Incorporating critical social sciences in education to ‘show that what managers do is fundamentally social, and is shaped by social boundaries, social relations, and power’ (Adams et al., 2011, p. 168).
4. Unifying the perception of SD among university interest groups, such as students, teachers and administrative staff, to ensure the adaption of the educational process to common goals.
5. Ensuring a desired level of awareness about SD and SDGs across all university subjects or units.

The socio-cultural environment also affects how sustainability is taught (Filho et al., 2018; Jónsson et al., 2021). In this current study, the focus is on sustainability education in Finland, which was the number one country in the UN SD ranking in 2021 (Sachs et al., 2021). However, despite being at the top in implementing SDGs, Finland still faces challenges in achieving some of the SDGs, for instance, SDG 12 ‘Responsible consumption and production’ and SDG 13 ‘Climate action’ (ibid.). Furthermore, developed countries, such as Finland, usually have a lower international spillover score, which means that they have a more negative spillover effect on the economies of other nations (Sachs et al., 2021). Therefore, when teaching sustainability in business in Finland, the focus should be on improvement regarding the SDGs that are challenging and should consider the international interdependence factor, given that ‘no business is an island’.

Conventionally, learning sustainability-related subjects may be approached from the constructivist learning theory perspective, which embraces interactive learning, where students co-create knowledge about SD challenges under the teachers’ guidance (e.g. Dziubaniuk & Nyholm, 2020). Another common theoretical approach—transformative learning—fills students with knowledge transferred from a teacher and demands critical assessment and interpretation of the obtained knowledge, which is especially significant for approaching sustainability (Rodríguez Aboytes & Barth, 2020; Seatter & Ceulemans, 2017). Novel disruptive learning theory concerns interventions to the students’ thoughts, making emotional reactions and mental connections stimulate their learning and, eventually, the internalisation of the learned experience regarding SD issues (Tillmanns, 2020). Overall, teaching sustainability may require constructivist, student-centred and transformative pedagogies, both in offline and online environments, to transform students’ attitudes and behaviours towards SD-related issues and concepts (Nousheen & Kalsoom, 2022). Conventional learning theories rarely regard the role of digital technologies in learning frameworks as enablers and sources of knowledge about SD, whereas the digitalisation of learning is currently in high demand.

The current environment and the more intensive switch to digital education may bring new challenges for educators. For instance, HEI educators may resist a shift to digitalisation of the study process due to reliance on conventional teaching methods, their lack of professional competences in digital tools or having experiences of using technology of poor quality (Bond et al., 2018; Kordrostami & Seitz, 2022; Mercader & Gairín, 2020). However, digital technologies have become more frequently applied to support educational programmes (Rof et al., 2020). In particular, digital technologies and methods may be applied to sustainability education in the form of, for instance, games and simulation, aiming to develop critical thinking or to explore challenges of sustainability in certain geographical areas (Bachen et al., 2015; Nilsson & Jakobsson, 2011). Technologies can be used to facilitate studies of climate change (Bush et al., 2016), entrepreneurship for sustainability (Rashid, 2019), engineering studies for sustainable solutions (Sivapalan et al., 2016) and financial studies to address SDGs (Mori, 2020). In general, technology enables teachers and students to access a wide variety of information online and in multiple formats. Students may customise their learning according to their learning style in terms of time, space and access to study materials (Henderson et al., 2017). Engagement with digital technologies for studying increases the autonomous performance of students in searching and processing information, which enhances their independence and learning ‘how to learn’ (ibid.). Online technologies can also be used to develop a platform for social media and students’ engagement in learning from each other as they already actively use social media for other purposes (Duffy & Ney, 2015; Sangrà & Wheeler, 2013). Although a few articles have discussed the challenges and benefits of teaching sustainability in an online environment (e.g. Ahel & Schirmer, 2023; Nousheen & Kalsoom, 2022), there is a need for clear theoretical approaches that will act as a base framework for teaching sustainability in this relatively new context.

It is important to apply learning theories to the investigation of students’ learning, considering the fast-changing learning environment in HEIs. Learning theories aim to describe the process of learning and conceptualise a framework for instructional design. They also aid educators in developing learning environments that allow students to obtain the most from their learning experiences (Grassian & Kaplowitz, 2009). Connectivism learning theory, which is the focus of this study, is discussed in the following section.

### Connectivism theory and digital learning

Connectivism theory was conceptualised in the 2000s and posited as a ‘learning theory for the digital age’ (Kop & Hill, 2008). Connectivism is a “*theory of learning which elucidates how the internet has generated different and varied chances for human beings to learn from the internet and from each other*” (Bharucha, 2018, p. 200). The development of digital technologies served as a trigger for rethinking HEI structures and how teaching and interaction occur between students and teachers and among students. Connectivism emphasises the role of digital technology in accessing multiple sources of information and the development of skills for the assessment of these sources in the information network (Dunaway, 2011; Utecht & Keller, 2019). Social media, massive online open courses, educational games, open educational resources and other digital novelties have brought a cultural change in values (Saykili, 2019) and roles. Thus, the role of a teacher shifts from that of a lecturer to that of a facilitator, who is there to assist in the more autonomous process of student learning (Goldie, 2016). Hence, the impact of a teacher’s affective engagement skills on the learning process is emphasised in the online environment (Kordrostami & Seitz, 2022).

According to connectivism, knowledge is developed when a learner makes mental connections between concepts, ideas and opinions that can be accessed via internet-enabling technologies, which makes information technologies an inevitable part of learning facilitation (Dunaway, 2011). The eight key principles of connectivism state that:

1. Learning and knowledge rest in the diversity of opinions;
2. Learning is a process of connecting specialised nodes or information sources;
3. Learning may reside in non-human appliances;
4. The capacity to know more is more critical than what is currently known;
5. Nurturing and maintaining connections is needed to facilitate continual learning;
6. The ability to see connections between fields, ideas and concepts is a core skill;
7. Accurate, up to date knowledge is the intent of all connectivist learning activities; and
8. Decision-making is in itself a learning process (Siemens, 2005).

The main idea of these principles is that a learner can create new meaning instead of memorising facts and that knowledge is not in the facts themselves but in the ability to learn, unlearn, and relearn information and be able to apply the knowledge in an everchanging information environment (Dunaway, 2011; Goldie, 2016; Utecht & Keller, 2019). According to this logic, learning is about discovering new concepts, unlearning is the critical assessment of previous information, and relearning embraces a new understanding and replacing old beliefs or experiences because of new information (Utecht & Keller, 2019).

Thus, connectivism allows students to develop the key competences that lay at the foundation of 21st-century teaching and learning, namely, critical thinking, collaboration, communication, creativity and innovation, self-direction, making global and local connections and using technology as a tool for learning (Niu et al., 2021). Within the framework of connectivism theory, learning occurs in a highly collaborative and interactive environment that is open to various perspectives and encourages individual autonomy (Thota, 2015). In particular, this is beneficial when solving challenges of sustainable development, which require collaborative sharing of experience, both in business and educational contexts (Takala & Korhonen-Yrjäheikki, 2019). The activities that affect learning when a connectivist approach is applied are as follows:

1. Aggregation, by gathering various accessible pieces of information and resources;
2. Relating, by reflecting on the new knowledge through the prism of the learner’s past experiences and knowledge;
3. Creation, which involves learners in developing knowledge output of their own (e.g. presentation, blog post and web-based discussion); and,
4. Sharing the created insights with others (Kop, 2011; Thota, 2015).

Connectivism is not without challenges and critique. Due to the high level of autonomy in learning, it requires the students to take the initiative, be creative and be innovative. Whereas some students gain from this level of autonomy, others, who are not as self-directed, may feel a lack of support and guidance, which in turn may hinder their learning process (Thota, 2015). The level of student self-direction may also vary across countries. Finland is among the top countries in terms of education in various rankings (OECD, 2022), and students have a high appreciation of self-directional values (Verkasalo et al., 1994). The students’ ability to think critically may also be a challenge when implementing connectivism theory, considering that the amount of information on the internet is tremendous and thus requires certain skills for selecting information and assessing its relevance and credibility; hence, the development of critical thinking is among the key capabilities of education for sustainability (Filho et al., 2018; Sammalisto et al., 2015). Regarding SD studies, the critical assessment of information aids students in interpreting facts according to their own understanding, which may also be affected by their social interactions with teachers and other students (Dziubaniuk & Nyholm, 2020). Although the application of connectivism theory in teaching may help develop critical thinking further, it requires a certain initial level of competence. Developing critical thinking skills in learners is a cornerstone of all levels of the Finnish education system (Horn & Veermans, 2019). Hence, Finnish students already have some level of these skills by the time they enter university.

Another challenge of connectivism theory that has previously been highlighted in the literature is presence, that is, when a student is able to “*experience the activity as if it was taking place in real life, without the mediation of the computer*” (Kop, 2011, p. 22). An enhanced depth of learning requires cognitive presence, social presence and teacher presence (ibid.). Applying connectivism theory does not always require the teacher to be present, whereas students can have a facilitator role. Increased social presence is also challenging, considering that it depends on “*the individuals’ perceptions of each other’s immediacy, intimacy and a sense of group cohesion*” (Akcaoglu & Lee, 2016, p. 1), which can be partly affected by the teacher but also depends on the students themselves and their previous experiences. These days, presence can also be enhanced using virtual reality (VR) technologies in teaching (Shin, 2017) and other novel digital tools. Furthermore, the Covid-19 pandemic acted as a shift for students in becoming more present and comfortable in an online environment. The increased utilisation of digital technologies due to the pandemic has also increased the focus of pedagogy researchers on connectivism theory as a framework for the organisation and management of learning (Al-Mutairi & Mubayrik, 2021).

The rapid disruption of the educational system and the switch to online learning and teaching made teachers worldwide adopt technologies to adapt the study process to the new reality (Pokhrel & Chhetri, 2021). The pandemic disruption revealed a gap in digital skills among educators and students and a demand for flexibility in studies and learning (Webb et al., 2021). Despite many challenges, such as anxiety and lack of training in the use of digital tools, the switch to online learning made educators to search for new technological solutions and digital platforms for the facilitation of learning (La Velle et al., 2020). This led to the creation of cooperative, interactive and flexible materials that prepare students for the digital world, which is also demanded in the labour market (Zawacki‐Richter, 2021). As the pandemic has slowed down, many results of the digitalisation of education remain in use because of their convenience and adaptability to the learning environment. This opens a discussion on the future of HEI learning in the digital era and how study processes can be organised to meet students’ needs for knowledge, among others, in the spheres of business and sustainability.

## Methodology

This qualitative study is based on an analysis of textual data in the form of course reflection essays. The reflections were written by the students who participated in the course Sustainable Business conducted at the subject International Marketing, ÅAU during the pandemic years 2020 and 2021. The objective of the course is to increase the participants’ knowledge about sustainability from a business and marketing perspective by assessing environmental, social and economic challenges. During the course, the students were expected to develop knowledge about how business organisations can respond to the demands for sustainable solutions set by customers, policymakers and other interest groups. In addition, the course discussed how businesses can be proactive in approaching new markets and creating competitive advantages through sustainability and corporate social responsibility (CSR).

Before the pandemic, the course was conducted in class with conventional teaching methods, such as lectures, seminars and home assignments. In addition to the individual work of writing essays about selected topics and analysing case studies, the course also included collaborative assignments done in groups, such as the analysis of companies’ sustainability initiatives and the development of a business plan for a company with sustainability at the core of its business model. In the spring of 2020, the pedagogics of the course were changed into online teaching. All interaction and knowledge transfer between the course instructors and the students occurred via Zoom, the course web page on the Moodle platform and emails. The teachers’ roles were to facilitate an efficient distant learning environment and to assess the students’ performances. A course reading package was available from the university e-library, and individual learning also took place via internet searches for relevant and current information. At the end of the course, the students had to provide free-form feedback and answer the following questions: (1) What are the most important things that you have learned, and how do they link to your previous knowledge and experience? (2) How can you benefit in the future from the things you learned during the course? (3) When and how can you put into practice the things you have learned?

The number of collected course evaluation essays was 71 (year 2020) and 95 (year 2021), 166 essays in total. These textual artefacts were analysed through the framework of content analysis (e.g. Duriau et al., 2007; Gibbs, 2018) and with the help of NVivo qualitative data analysis software (e.g. Jackson & Bazeley, 2019). For the structural data analysis, each student’s essay obtained an index number in accordance with the year it was written (e.g. 1:2020—essay number 1, year 2020). The aim of the analysis was to identify how students make connections among the concepts that they have learned and how they transform these into knowledge that follows the principles of connectivism. The essays were read and analysed by the researchers, of which two were the main instructors of the course. The involvement of several researchers in the analysis had several benefits related to investigator triangulation (Flick, 2004): it allows for comparison of the results, increases study trustworthiness and helps in avoiding bias of the researchers involved in the course teaching. In the first step of the analysis, the essays were read in search of expressions and statements reflecting the learned concepts about SD, descriptions of the learning process, links between the created knowledge and the students’ main field of study, descriptions of practical assignments as learning tools, group and individual work and critical assessment of the digital learning. The relevant pieces of text were grouped according to textual codes created in NVivo based on the principles of connectivism. The main codes included sustainability perspective (student’s understanding of SD concepts), learning process (how information nodes were linked in the students’ understanding), technologies (reflection on digital technologies as enablers of learning and communication), critical thinking (ability to assess information), making connections (ability to develop communication skills and facilitate individual learning), links (ability to make conclusions of the learned information), new learning (ability to continue individual learning) and decision making (influence of SD knowledge on decision making on students’ daily lives, consumer behaviours, business decisions and careers). The interpreted data are presented in the Results section. Quotations from the students’ reflections were used to support the structural presentation of the findings.

## Results

The results are presented according to the eight main principles of connectivism formulated by Siemens (2007). Since connectivism is a framework for understanding the learning process (Dunaway, 2011), the focus is set on the students’ learning experience, concepts they learned during the course and connections they made between concepts, opinions and various perspectives of the obtained knowledge.

### Learning and knowledge rest in the diversity of opinions

The students emphasised that the course allowed them to approach sustainability from different perspectives and that the business perspective became especially eye-opening for many. Some students had previous knowledge, mostly about environmental sustainability. To this knowledge, this course added social and economic sustainability as a background for the triple bottom line (e.g. Elkington, 1997). Other frequently mentioned concepts concerned circular economy, business ethics, sustainability reporting, CSR, sustainable marketing strategies and greenwashing. According to the reflections, the course contributed to a re-learning of the basic understanding of sustainability, as expressed in the following: “*I used to be sure that business is a survival game, where everyone is just for himself, and the others do not care. However, having studied this course, it became obvious that working for the good of society is much more profitable than thinking only about your own profit*” (7:2020). Some students obtained new insights into previous knowledge after the course: “*I realized that my thoughts on sustainability and sustainable business were partly outdated. The course provided me (with) new insights on the progress made in sustainability and the companies’ growing efforts to act more sustainable and include sustainability in their business strategies*” (37:2021). Given that the course brought together students of very different subjects, such as business management, marketing, accounting, marine biology, chemistry and engineering, they extended their knowledge in fields previously unknown to them. For instance, students of the natural sciences developed an understanding of business and marketing. This knowledge may be useful in the future for their careers and their focus on the development of innovations, for instance: “*I am studying to become an engineer in the cosmetics industries. The concept of sustainability will be useful by innovating in sustainable packaging or proposing products promoting a plastic-free* w*orld”* (1:2020). Similarly, marketing students got deeper insights into sustainability reporting, CSR, accounting and business ethics.

The diversity of opinions is at the core of connectivism (Utecht & Keller, 2019) and played a significant role in knowledge development during the course. Through discussion of assignments in groups, students could learn about the opinions of others: “*To look at issues from a broader perspective rather than only from one is really refreshing*” (17:2020). For instance, the group assignment to develop a business plan for a sustainable company was successful in facilitating discussion, as indicated: “*The group assignment ‘Games of sustainable minds’ was a creative way to teach—or even better, let us to teach each other*” (25:2020). A diversity of insights also came from international students who included cultural perspectives on sustainability from different parts of the world. In addition, international students could learn from their Finnish peers and explore sustainability practices in Finland and Nordic countries. During the pandemic, all student discussions were conducted online, mostly via Zoom, which allowed the students to enhance their digital communication skills as they “*did a lot of Zooms, where we exchanged our opinions during our two group works, and it was very enriching*” (50:2021).

Another diversity of opinions came from access to online information. Online sources offer novel information in various forms. The students not only read academic and business articles but could also access podcasts or documentary movies (for instance, about textile recycling) that were not used in the conventional version of the course. However, the diversity of information sources online has its drawbacks, as information can easily be manipulated on the internet (e.g. Carusi, 2011). Therefore, critical assessment of the information was something that the students had to learn (Niu et al., 2021). For instance, students could explore how the good intentions of the companies ended up in greenwashing accusations for misleading marketing messages. In addition, the course instructions encouraged the students to use various information resources but prohibited referencing to Wikipedia.

Teachers also make input into the diversity of opinions as they lead and organise the learning. A teacher’s role is to direct students onto the right path and motivate them to continue learning even after the course (Dziubaniuk & Nyholm, 2020). The presence of a teacher or instructor during distance learning provides a feeling of community and belonging to the social group (Akcaoglu & Lee, 2016). Information technologies in this case came to the stage as enablers of the learning routine, organisers of the study process and tasks managers (Henderson et al., 2017). An important task of the technologies is to facilitate communication between teachers and students, especially when face-to-face interactions are not possible. However, despite best efforts of organising online infrastructure for the course, not everyone remained happy about it: “*Something that annoyed me throughout the course was the quite honestly annoying layout of the Moodle page*” (58:2020).

### Learning is a process of connecting specialised information sources

Connectivism assumes that a learner makes logical connections between concepts, ideas and opinions into a network of knowledge (Dunaway, 2011; Niu et al., 2021). Linking new and previous knowledge is at the core of the learning process in this case: “*These various important topics allowed me to make the link with my previous knowledge and experiences. Being a student in management and now in marketing, I already had the basics of business, but I wanted to add the sustainable side because I think that it is essential to be able to deal with the issue of perspectives of a company’s sustainability work*” (50:2020). According to the students’ reflections, they managed to make links between knowledge about sustainability and business and connect this knowledge with their main study subjects that might have no relation to SD or business. This indicates that students will be able to use this knowledge in their future employment, as well as in their daily lives. Some reflections indicated that the learned principles of sustainability can be implemented to change daily routines towards more sustainable lifestyles (e.g. more engagement in recycling, eliminating food waste and using less plastic products) and change the behaviours of the students as consumers: “*I have now started to look for alternative option when buying clothes and food*” (53:2020) or “*During the course, I have begun to look more closely on what I buy and how I can affect as a consumer*” (45:2021).

Several students mentioned that they intend to start their own businesses in the future, and they could see how sustainability could be a vital part of their business strategy. To make such connections, students had to combine knowledge obtained from the articles, social media, forums and discussions with each other at the seminars hosted via Zoom and through analysis of the companies’ websites and related media material. Technologies, in this case, provided flexible access to various information sources, whereas course instructors led the learning in the right direction through thematic lectures, assignments and seminars.

### Learning may reside in non-human appliances

Digital technologies develop learning skills for searching for information and for critical assessment of data sources. This especially concerns the critical assessment of social media, which is one of the digital literacy skills (Utecht & Keller, 2019). Students learn how to search for data and how to filter information in search engines in order to obtain the most relevant pieces. Fast access to information has become a new normal that directly influences learning (Saykili, 2019). Thus, courses facilitated by technology increase students’ skills in searching for and critically processing information. As indicated in an essay, “*I have truly found some good [online] resources for gathering new information on the subject and will continue to study*” (2:2020). Searching and processing information develop analytical skills, leading to critical assessment (Niu et al., 2021) and, as pointed out by the students, “*Today, we have massive amounts of data that show how much any industry pollutes []*” (13:2020) or “*I can make analyses of a company by performing a review of their corporate social responsibility, green supply chain, *etc*.*” (14:2020).

Technology proves to be efficient for flexible information access, as indicated: “*I could not follow all the guest lectures but reading the material on Moodle I found the topics compelling and worth to examine in more depth*” (4:2020). Recorded lectures may also be useful for refreshing what was said during the classes and in case a student has missed some sessions: “*I haven’t been able to participate a lot on those discussions in Zoom (because of my work), but I have learned new things from the videos afterwards*” (25:2020). Technology also adds convenience to the completion of assignments: “*Having virtual meetings, doing assignments together virtually saved a lot of time and effort on planning and discussing matters*” (32:2020). Along with managing information, the students also enhanced their skills in using software: “*I will be taking with me from this course, very unexpectedly, is the knowledge of how to use Adobe’s video editing programs*” (93:2021). Mastering hardware, software and individual work at a distance will be beneficial for the students’ future work careers, as these skills are expected in modern job markets: “*On a personal level, one of the most important things I learned in this course is the ability to work together with other people and how teamwork works (remotely)*” (66:2021).

Despite the usefulness and flexibility of technology, the rapid shift to distance education can be difficult, not only for teachers (Bond et al., 2018; Mercader & Gairín, 2020) but also for some students, as indicated: “*To be honest, I miss normal lectures and normal university-days*” (20:2020); or “*In my opinion, this course would have been better if we have had possibility to see each other face to face and discuss or even debate on sustainability aspects”* (25:2020). Evidently, online learning is not a solution that fits everyone. A hybrid mode of teaching or blended learning can be a solution when some presence in class is possible (Dziuban et al., 2018; Galvis, 2018). Within the scope of online teaching, students can be allowed to personalise their learning with more individual assignments so that they can choose the time and place for their own learning.

### Capacity to know more is more critical than what is currently known

Learning is more crucial than knowing (Siemens, 2007). This statement embraces the idea that unlearning and relearning provide a new critical understanding of reality compared to only knowing some facts (Utecht & Keller, 2019). Information on the internet is updated every day, with new information being added to the information landscape. Searching, extracting and processing relevant information have become important learning skills. To continue learning about sustainability and business subjects, students need to understand what keywords to use in search engines. Thus, concepts introduced during the course are useful not only for their meaning as such, but also for continued learning after the course. For instance, the most common concept mentioned in the reflection essays was greenwashing. Greenwashing can be understood from different perspectives, from misleading marketing campaigns to the fast flow of financial benefits to the company due to unethical business management. According to the reflection essays, the students managed to make a mental connection between the different aspects of greenwashing by analysing examples of companies’ marketing campaigns and CSR reports. Possessing this knowledge, they can also more critically assess marketing messages in their role as consumers.

The critical assessment of information is also important for the development of critical thinking (Niu et al., 2021). Critical thinking regarding SD and business allows one to explore these subjects from various perspectives (Filho et al., 2018). For instance, students may develop a different understanding of how SD can be implemented in their home countries in comparison to others with different socio-economic backgrounds. Pedagogically, this learning was supported by discussions with international students at the seminars, during the preparation of group assignments and when analysing companies’ sustainability reporting referring to academic literature. According to the reflection essays, the students’ critical thinking developed concerning marketing programmes, consumer behaviour and eco-labels, being “*critical towards business models*” (8:2020), “*companies’ sustainable measurement*” (19:2020), “*products which are marketed as green*” (23:2020), “*advertisements of the companies*” (69:2021) and critical “*ability to distinguish words from deeds in a company’s sustainability*” (15:2021).

### Nurturing and maintaining connections is needed to facilitate continuous learning

Connectivism proposes that learning is a result of collaboration (Siemens, 2005). Collaboration in this case occurs not only in direct student interaction, but in time and space (Utecht & Keller, 2019). Online courses are accessible from various places and at various times, and students may complete their group assignments by sharing text-based files (e.g. in Google Docs). Despite social distance, the students found that “*working in groups was already a good source of learning*” (52:2020). During the course, the students participated in case discussions led by the instructors via Zoom and in the preparation of group assignments, such as company sustainability analysis and the development of a company business plan. Therefore, once again, the students had a chance to enhance their social and online communication skills: “*This course has once again improved my networking skills and the ability to cooperate in groups. Working in groups is something that I will get used to in the future, which is why it is so important to practice interacting with people already at an early age*” (54:2020). Another learning point was that the groups consisted of students representing different subjects, so “*while doing projects we find that we have quite different styles to think about things but that was just a strength for our group*” (51:2020).

### Ability to see connections between fields, ideas and concepts is a core skill

The ability to find links between ideas and concepts requires research skills and creativity in thinking that make students active learners (Niu et al., 2021; Siemens, 2005). Students should be able to create new meanings from those connections through, in this case, individually analysing companies’ actions, marketing communication and sustainability reporting. Regarding this course, creativity was needed, particularly for the development of a business plan, when students in groups had to come up with business ideas for sustainable companies. This assignment allowed them to share information, learn from each other during discussions and develop a business plan based on theory and practical examples of companies they found on the internet. As noted by the students, “*this assignment was nice to be challenged creatively and think about a functioning business model with sustainability as the key element*” (27:2020), or “*individual assignments were more reflective, and the group assignments were more creative and fun to do as a team*” (42:2021). Working on such assignments can be at the core of connectivism, where individual learning is combined with information sharing and creativity for the creation of new meaning.

Regarding the business perspective, it can be concluded that the students made links between the companies’ sustainability actions and their performance in the markets. Ethical companies acting responsibly are considered to be those that have more chances to stay profitable in the long run. Connections were also made between the marketing communication of business organisations through CSR reporting, sustainability labelling, certificates, charity programmes and the companies’ image and reputation.

### Accurate, up-to-date knowledge is the intent of all connectivism learning activities

Connectivism requires two skills for the facilitation of learning: the ability to search for current information and to filter secondary or extraneous information (Melrose et al., 2013). In comparison to the first principle, where learning comes from a variety of sources and opinions (Siemens, 2005), this principle highlights how a person can keep their knowledge accurate and up-to-date in the modern information-rich world (Utecht & Keller, 2019). In this case, the volume of information grows about, for instance, companies’ sustainability initiatives, sustainable innovations and the EU’s policies on SD. The ability to search for and process current information becomes vital. The accuracy of information also becomes critical, as does requiring the skills to recognise if the information is trustworthy.

Critical thinking comes to help in this case, and that is what the course aims to develop. For instance, the students indicated that they critically evaluated the CSR reports of the companies that they analysed in groups. In addition, in their final course essays and in the group assignments, they criticised the information that companies posted online about sustainability initiatives, as this information can be manipulated for marketing reasons and can be difficult to verify. The course illustrated the importance of using search engines for data analysis, finding information that may not be advertised by the company and referring to different information sources.

### Decision making is in itself a learning process

In the realm of the changing reality and information flow, decisions should be made on what to learn, from whom and whether this knowledge is trustworthy. Knowledge that is right today may become wrong tomorrow because of changes in the information landscape affecting decisions (Siemens, 2005). As indicated in one student’s essay, “*there are no one single correct answers, but one has to build one’s own truth* [about sustainability in business]” (2:2020). By following changes in legislation regarding SD, consumer market trends and reactive companies’ actions towards SD, the students developed the understanding that information and reality are changing all the time and that continued learning and critical assessment of information are required for making the right decisions in business management and consumption. This may concern, for instance, updates to governmental policies on SD in different countries. This knowledge is important from a business perspective, as changed regulations may impact decisions about business strategy development.

The course instructors led the students towards learning by structuring the course content so that the students could later decide on what information to continue learning about and from where this information should be obtained. Thus, decision making is a learning process as it involves previous knowledge and assessment of current information. From the business perspective, students seem to understand that *“[sustainability] is surely a factor that must be considered in decision making [at the companies’ management]*” (64:2020) and that “*companies have the potential to impact societies and the environment both negatively and positively. Thus, it is crucial that they consider all aspects of sustainability in their decision-making*” (64:2021). Up-to-date information available online and knowledge about sustainability best practices obtained from the course content have become necessary for supporting managerial decisions. In addition, a decision to change a daily lifestyle towards a more sustainable one, as pointed out previously, is also both a process and an outcome of learning. The environment is constantly changing by offering more sustainable solutions and by shifting consumer behaviour. Students should be able to recognise these trends and make decisions about adopting them in their professional and daily lives.

## Discussion

According to the results of this study, the students were able to develop their knowledge and understanding of SD in the business field by making links between concepts, such as the conventional meaning of sustainability (as stated in Brundtland’s report, 1987) and practical cases of sustainability initiatives introduced by the companies and understanding how these initiatives affect stakeholders. Among other concepts, the students distinguished the principles of circular economy, CSR, sustainability reporting, misleading marketing (greenwashing), ‘green’ supply chains and ethical business conduct. They also connected the learned concepts about sustainability to their major studies, future careers, daily lives and consumer behaviours. In addition, they have reflected on how SD may be important for business management in terms of new business models, entrepreneurship or transition towards sustainability, which are among the main learning objectives of the course. Painter-Morland et al. (2016) framed these learning outcomes as capacity building in students who develop their skills and knowledge for SD. Making mental connections is among the key principles of the development of learning from the connectivism perspective (Siemens, 2007). This way of learning adds new knowledge to the previous one and allows for relearning and unlearning concepts that were previously understood as the only truth (Utecht & Keller, 2019). For many students, making connections between sustainability concepts was ‘eye opening’, as they developed an understanding of how SD affects the world and their lives as well. In this case, the teacher plays the role of a facilitator, assisting in the student’s learning (Goldie, 2016). However, principles of connectivism also allow students to take a leading role in the learning process (Akcaoglu & Lee, 2016; Thota, 2015) that may help them to develop independence in learning but may also be a hindering factor for those who prefer learning in ‘real-life’ environments.

Social interaction with the help of digital technologies is becoming mainstream in business life, especially after forced distance work and education during the pandemic (e.g. Pokhrel & Chhetri, 2021). Thus, the adoption of online communication technologies helped students not only to access up-to-date current information but also to increase their skills in managing digital technologies and information. An important learning outcome was the increased ability to manage social networking online for the accomplishment of assignments. This skill may be useful in the future for conducting and managing international business or social projects (where interaction occurs between different places and time zones), given that projects for sustainability tend to engage many stakeholders on an international scale (e.g. Dziubaniuk et al., 2022; Romestant, 2020). Recorded lectures, students’ presentation videos and a reading package available in the digital format of the course helped to achieve flexibility in accessing the educational materials in time and space—a gap that became visible during the pandemic (Webb et al., 2021). Access to the created study materials at any time, including during online streamed lectures and seminars, allows students to customise the learning process, which may reflect positively on their learning, as indicated by Henderson et al. (2017).

Learning also occurred in student groups and during the writing of individual assignments. Groupwork, as a form of connective learning, supported the students’ learning from each other. In addition, virtual groupworks also facilitated informal interaction between the students and teachers, making this similar to the social media interaction that has become natural in the modern digital age (Duffy & Ney, 2015; Sangrà & Wheeler, 2013). Working in teams enhanced the students’ learning, especially when the groups consisted of international students representing various cultures. The socio-cultural context of an HEI may shape the methods of how sustainability is represented in the curriculum (Filho et al., 2018), but various opinions and cultural perspectives on SD remain important learning outcomes. In this case, the cultural mix of students provided for various insights into sustainability and the business practices in various countries, which are crucial for future work in companies that have to account for various perspectives on sustainability when dealing in, for instance, emerging countries (Ivanova-Gongne et al., 2022).

The individual tasks during the course were based on writing assignments that required the search and analysis of online information. These assignments demanded critical thinking to recognise relevant knowledge in the sea of available information on the internet, where information can easily be manipulated (Carusi, 2011; Utecht & Keller, 2019). Critical thinking, as one of the key principles of connectivism (e.g. Goldie, 2016; Siemens, 2007), is also necessary for the analysis of misleading marketing approaches (greenwashing) and unethical behaviour of international companies. Critical assessment and experience are also significant for decision making as a learning process (Siemens, 2005) concerning sustainable consumption and the management of business processes. This is in line with Adams et al.’s (2011) discussion of the challenges of teaching sustainability and, specifically, the development of critical self-reflection skills that eventually would be reflected in managerial decision making.

In addition to the knowledge of sustainability in business obtained during the course, the students also improved their skills in managing technological advancement: making video presentations, pitching business ideas online, organising virtual communication for their project work and making online presentations. These skills are vital in the digital age and will be even more demanded in the future, since online-enabling technology is not only a collaborative method of communication but also a tool of learning and developing knowledge (Niu et al., 2021). Notably, the students were encouraged to expand their learning beyond the course requirements by watching recommended documentaries about, for instance, sustainable textile recycling or listening to podcasts about SD topics that are easily accessible online. Such a need for current information retrieval is related to the everchanging informational environment (Melrose et al., 2013) and a necessity to keep up with sustainability trends that affect not only business strategies but also social lives. Access to information from different sources, the ability to see the connections between these and nurturing these connections also encourage students to continue learning, which is among the key principles of connectivism (Siemens, 2005).

Despite several pedagogic benefits, connectivism is still criticised for lacking theoretical borders and being a pedagogic approach rather than a learning theory (Oommen, 2020; Verhagen, 2006). Despite this critique, connectivism offers a framework for understanding learning in the modern context of digital learning spaces (Dunaway, 2011). This learning theory allows us to frame knowledge generation from the internet and interactive learning from social networking facilitated online (Bharucha, 2018). However, face-to-face interaction among students and teachers remains irreplaceable despite all the benefits and flexibility of digital learning, as indicated in some of the student diaries. The full digitalisation of HEI courses is possible and can be utilised during forced social distancing (e.g. during pandemic situations or the necessity of knowledge dissemination over time and space). The digitalisation of communication can be used as an enabler of learning but should not completely replace live social interactions in universities. Hybrid or blended learning modes (combining sessions in the classroom and online) seem to be a better solution (e.g. Dziuban et al., 2018; King & Sizemore, 2020; Skulmowski & Rey, 2020).

## Conclusion

Sustainability has become a vital part of the university curriculum for educating future business leaders and entrepreneurs. Teaching sustainability is related to building capacity in students by developing their skills concerning SD. Organising such a learning process requires modern and transformative approaches, not only in terms of technology usage, but also new conceptual approaches to the facilitation of learning. The connectivism learning theory presents opportunities for educators to structure courses on business and sustainability in efficient ways at an HEI. This learning approach builds on the utilisation of digital technologies that connect students to up-to-date information and to each other and on technologies that help instructors to structurally organise the study process. In particular, this concerns courses dedicated to sustainability in business, where it is important to refer to up-to-date information, such as business cases, sustainability trends, governmental regulations and international policies. This study also emphasises that knowledge is not a set of facts about a subject but the accuracy and speed with which one can learn, unlearn and relearn information. Thus, connectivism, as a learning framework, can be considered for the development of university curriculum regarding SD, and business studies as technologies facilitating learning have become a vital part of the educational process and will play an even more significant role in the future.

### Implications

Regarding theoretical contributions, this study adds to the literature by covering issues of education for SD and pedagogic practices for sustainability (Kopnina, 2020; Painter-Morland et al., 2016). This study highlights the importance of teaching sustainability in the context of business and marketing studies to illustrate the responsibilities that business organisations and related stakeholders bear towards nature and society. In addition, this research denotes that pedagogic attempts should introduce SD not as a burden to business organisations but as a market opportunity and a way to reach economic stability.

This study extends the knowledge on connectivism learning theory by demonstrating its empirical application to the scope of the course on sustainability in business (Abad-Segura et al., 2020; Gómez-Zermeño, 2020; Oommen, 2020). Although there have been a few studies on educating sustainability in an online environment (Ahel & Schirmer, 2023; Nousheen & Kalsoom, 2022), they still lack a solid theoretical approach to support the pedagogic design of such courses. This empirical research shows that connectivism is a relevant theoretical perspective to enable a transformative approach to teaching, which is crucial for learning sustainability (Nousheen & Kalsoom, 2022). Utilising novel learning theories as frameworks for designing HEI courses is important in the digital era and for students’ perceptions of constantly changing information influenced by technologies (Bond et al., 2018; Waycott et al., 2010). Distant and blended modes of education have gained popularity due to their flexibility and inclusivity (e.g. Dziuban et al., 2018; Skulmowski & Rey, 2020). However, they require novel approaches to the planning of teaching and more research on how to make such learning efficient.

### Limitations and recommendations for future research

This study has several limitations. First, the research is focused on a specific course dedicated to sustainability in businesses and undertaken in the context of a HEI located in Finland. The country context may have a socio-cultural influence on how university courses are structured and on their content. However, the Finnish context is a suitable case for study considering the country’s policies focused on SD and its international collaboration for sustainability (Jónsson et al., 2021; Ministry of Education & Culture, 2020). Finnish universities have relative freedom in curriculum planning that does not limit instructors’ creativity in designing courses. Nevertheless, this study calls for further research on SD-related pedagogic methods applied to courses conducted in the context of HEIs. Novel pedagogic approaches can focus on cross-cultural perspectives in course design due to the large number of international and exchange students in European HEIs and the complexity of the sustainability concept in an international environment. Referring to the cross-cultural perspective in course design would allow students to learn about the diverse perceptions and understandings of sustainability in various cultural contexts, which is crucial when operating on global markets and in international business relationships (Ivanova-Gongne et al., 2022). Globalisation and social challenges in the business sphere also need to be approached through efficient teaching methods. More research is also needed to address challenges about innovative and experimental digital technologies, such as the use of VR technology (Shin, 2017), for facilitating teaching and learning processes in an HEI, that also may require appropriate ontological and epistemological approaches to the students’ learning and information appropriation.

Second, this study is based on empirical data collected during two years of the Covid-19 pandemic, whereas a longitudinal study would allow for the collection of more course reflection essays. This also shows a need for more studies addressing the adaptation of university learning environments to rapid societal changes. This study can also be useful for pedagogy studies applied in a digital environment to to gain more insights into best practices and creative teaching methods developed during the distance learning period and adopted in post-pandemic times.

Third, given the focus on the students’ reflection essays, this research mostly adopted the students’ perspectives on learning sustainability in business in the digital age. The teachers’ role was only partially covered in relation to the connectivism approach. Considering that course instructors have the role of learning facilitators, their perspective should be investigated in future research to address various methods for guiding knowledge development and frameworks these teachers apply when designing the study units.

## Data Availability

The qualitative dataset analysed in this study is available from the corresponding author upon reasonable request.

## Abbreviations

CSR: Corporate social responsibility
HEI: Higher education institution
SD: Sustainable development
SDG: Sustainable development goal
VR: Virtual technology

## References

[CR1] Abad-Segura E, González-Zamar MD, Infante-Moro JC, Ruipérez García G (2020). Sustainable management of digital transformation in higher education: Global research trends. Sustainability.

[CR2] Adams CA, Heijltjes MG, Jack G, Marjoribanks T, Powell M (2011). The development of leaders able to respond to climate change and sustainability challenges: The role of business schools. Sustainability Accounting, Management and Policy Journal.

[CR3] Aggarwal R, Wu Y (2020). Online teaching in international business. Journal of Teaching in International Business.

[CR4] Ahel O, Schirmer M (2023). Education for sustainable development through research-based learning in an online environment. International Journal of Sustainability in Higher Education.

[CR5] Åbo Akademi (2020). Strategi 2021–2030. https://www.abo.fi/wp-content/uploads/2020/03/AA-strategi-2020.pdf. Accessed 15 May 2022.

[CR6] Akcaoglu M, Lee E (2016). Increasing social presence in online learning through small group discussions. The International Review of Research in Open and Distributed Learning.

[CR7] AlDahdouh AA (2018). Jumping from one resource to another: How do students navigate learning networks?. International Journal of Educational Technology in Higher Education.

[CR8] Al-Mutairi NM, Mubayrik HFB (2021). Connectivism learning theory to enhance higher education in the context of COVID-19 pandemic. International Journal of Educational Sciences.

[CR9] Anastasiadis S, Perkiss S, Dean BA, Bayerlein L, Gonzalez-Perez MA, Wersun A, Acosta P, Jun H, Gibbons B (2020). Teaching sustainability: Complexity and compromises. Journal of Applied Research in Higher Education.

[CR10] Bachen C, Hernández-Ramos P, Raphael C, Waldron A (2015). Civic play and civic gaps: Can life simulation games advance educational equity?. Journal of Information Technology and Politics.

[CR11] Bagur-Femenías L, Buil-Fabrega M, Aznar JP (2020). Teaching digital natives to acquire competences for sustainable development. International Journal of Sustainability in Higher Education.

[CR12] Barnett J, McPherson V, Sandieson RM (2013). Connected teaching and learning: The uses and implications of connectivism in an online class. Australasian Journal of Educational Technology.

[CR13] Bharucha J (2018). Exploring education-related use of social media: Business students perspectives in a changing India. Education+ Training.

[CR14] Bien C, Sassen R (2020). Sensemaking of a sustainability transition by higher education institution leaders. Journal of Cleaner Production.

[CR15] Bond M, Marín VI, Dolch C, Bedenlier S, Zawacki-Richter O (2018). Digital transformation in German higher education: Student and teacher perceptions and usage of digital media. International Journal of Educational Technology in Higher Education.

[CR16] Bush D, Sieber R, Seiler G, Chandler M (2016). The teaching of anthropogenic climate change and earth science via technology-enabled inquiry education. Journal of Geoscience Education.

[CR17] Carusi A, Ess C, Thorseth M (2011). Trust in the virtual/physical interworld. Trust and virtual worlds: contemporary perspectives.

[CR18] Claro PB, Esteves NR (2021). Teaching sustainability-oriented capabilities using active learning approach. International Journal of Sustainability in Higher Education.

[CR19] Corbett F, Spinello E (2020). Connectivism and leadership: Harnessing a learning theory for the digital age to redefine leadership in the twenty-first century. Heliyon.

[CR20] Davidson J, Prahalad V, Harwood A (2021). Design precepts for online experiential learning programs to address wicked sustainability problems. Journal of Geography in Higher Education.

[CR21] Duffy K, Ney J (2015). Exploring the divides among students, educators, and practitioners in the use of digital media as a pedagogical tool. Journal of Marketing Education.

[CR22] Dunaway MK (2011). Connectivism learning theory and pedagogical practice for networked information landscapes. Reference Services Review.

[CR23] Duriau VJ, Reger RK, Pfarrer MD (2007). A content analysis of the content analysis literature in organization studies: Research themes, data sources, and methodological refinements. Organizational Research Methods.

[CR24] Dziuban C, Graham CR, Moskal PD, Norberg A, Sicilia N (2018). Blended learning: The new normal and emerging technologies. International Journal of Educational Technology in Higher Education.

[CR25] Dziubaniuk O, Ivanova-Gongne M, Berdysheva E (2022). Challenges of network interaction in managing sustainable development projects in developing countries: Case of an international consulting company. Critical Perspectives on International Business.

[CR26] Dziubaniuk O, Nyholm M (2020). Constructivist approach in teaching sustainability and business ethics: A case study. International Journal of Sustainability in Higher Education.

[CR27] Elkington J (1997). Cannibals with forks: The triple bottom line of 21st century business.

[CR28] Filho LW (2020). Accelerating the implementation of the SDGs. International Journal of Sustainability in Higher Education.

[CR29] Filho LW, Raath S, Lazzarini B, Vargas VR, de Souza L, Anholon R, Quelhas OLG, Haddadg R, Klavinsh M, Orlovic VL (2018). The role of transformation in learning and education for sustainability. Journal of Cleaner Production.

[CR30] Flick U, Flick U, von Kardorff E, Steinke I (2004). Triangulation in qualitative research. A companion to qualitative research.

[CR31] Friman M, Schreiber D, Syrjänen R, Kokkonen E, Mutanen A, Salminen J (2018). Steering sustainable development in higher education—Outcomes from Brazil and Finland. Journal of Cleaner Production.

[CR32] Gallagher S (2018). Development education on a massive scale: Evaluation and reflections on a massive open online course on sustainable development. Policy and Practice: A Development Education Review.

[CR33] Galvis ÁH (2018). Supporting decision-making processes on blended learning in higher education: Literature and good practices review. International Journal of Educational Technology in Higher Education.

[CR34] Gibbs G (2018). Analyzing qualitative data.

[CR35] Goldie JGS (2016). Connectivism: A knowledge learning theory for the digital age?. Medical Teacher.

[CR36] Gómez-Zermeño MG (2020). Massive open online courses as a digital learning strategy of education for sustainable development. Journal of Sustainable Development of Energy, Water and Environment Systems.

[CR37] Grassian E, Kaplowitz J (2009). Information literacy instruction: Theory and practice.

[CR38] Henderson M, Selwyn N, Aston R (2017). What works and why? Student perceptions of ‘useful’ digital technology in university teaching and learning. Studies in Higher Education.

[CR39] Horn S, Veermans K (2019). Critical thinking efficacy and transfer skills defend against ‘fake news’ at an international school in Finland. Journal of Research in International Education.

[CR40] Ivanova-Gongne M, Torkkeli L, Hannibal M, Uzhegova M, Barner-Rasmussen W, Dziubaniuk O, Kulkov I (2022). Cultural sensemaking of corporate social responsibility: A dyadic view of Russian-Finnish business relationships. Industrial Marketing Management.

[CR41] Jackson K, Bazeley P (2019). Qualitative data analysis with NVivo.

[CR42] Janoušková S, Hák T, Nečas V, Moldan B (2019). Sustainable development—A poorly communicated concept by mass media. Another challenge for SDGs?. Sustainability.

[CR43] Jónsson, Ó. P., Guðmundsson, B., Øyehaug, A. B., & Didham, R. J. (2021). *Mapping education for sustainability in the Nordic countries*. Nordic Council of Ministers, Copenhagen, Denmark.

[CR44] Kardes I (2020). Increasing classroom engagement in international business courses via digital technology. Journal of Teaching in International Business.

[CR45] Karlusch A, Sachsenhofer W, Reinsberger K (2018). Educating for the development of sustainable business models: Designing and delivering a course to foster creativity. Journal of Cleaner Production.

[CR46] Keen M, Brown VA, Dyball R, Keen M, Brown VA, Dyball R (2005). Social learning: A new approach to environmental management. Social learning in environmental management.

[CR47] King DR, Sizemore AE, Baumann S (2020). Strategic management in online and hybrid courses. Teaching strategic management.

[CR48] Klašnja-Milićević A, Ivanović M (2021). E-learning personalization systems and sustainable education. Sustainability.

[CR49] Kop R (2011). The challenges to connectivist learning on open online networks: Learning experiences during a massive open online course. International Review of Research in Open and Distributed Learning.

[CR50] Kop R, Hill A (2008). Connectivism: Learning theory of the future or vestige of the past?. International Review of Research in Open and Distributed Learning.

[CR51] Kopnina H (2020). Education for the future? Critical evaluation of education for sustainable development goals. The Journal of Environmental Education.

[CR52] Kordrostami M, Seitz V (2022). Faculty online competence and student affective engagement in online learning. Marketing Education Review.

[CR53] La Velle L, Newman S, Montgomery C, Hyatt D (2020). Initial teacher education in England and the Covid-19 pandemic: Challenges and opportunities. Journal of Education for Teaching.

[CR54] Lozano R, Ceulemans K, Seatter CS (2015). Teaching organisational change management for sustainability: Designing and delivering a course at the University of Leeds to better prepare future sustainability change agents. Journal of Cleaner Production.

[CR55] Lozano R, Young W (2013). Assessing sustainability in university curricula: Exploring the influence of student numbers and course credits. Journal of Cleaner Production.

[CR56] Manna V, Rombach M, Dean D, Rennie HG (2022). A design thinking approach to teaching sustainability. Journal of Marketing Education.

[CR57] Melrose S, Park C, Perry B (2013). Teaching health professionals online: Frameworks and strategies.

[CR58] Mercader C, Gairín J (2020). University teachers' perception of barriers to the use of digital technologies: The importance of the academic discipline. International Journal of Educational Technology in Higher Education.

[CR59] Merritt E, Hale A, Archambault L (2018). Changes in pre-service teachers’ values, sense of agency, motivation and consumption practices: A case study of an education for sustainability course. Sustainability.

[CR60] Ministry of Education and Culture (2020). *Sustainable development policy of the Ministry of Education and Culture and its administrative branch*. https://julkaisut.valtioneuvosto.fi/handle/10024/162185. Accessed 28 July 2022.

[CR61] Montiel I, Delgado-Ceballos J, Ortiz-de-Mandojana N, Antolin-Lopez R (2020). New ways of teaching: Using technology and mobile apps to educate on societal grand challenges. Journal of Business Ethics.

[CR62] Mori M (2020). Technology-enhanced financial education and sustainability goals. International conference on computer science, engineering and education applications.

[CR63] Mulà I, Tilbury D (2009). A United Nations decade of education for sustainable development (2005–14): What difference will it make?. Journal of Education for Sustainable Development.

[CR64] Nilsson EM, Jakobsson A (2011). Simulated sustainable societies: Students’ reflections on creating future cities in computer games. Journal of Science Education and Technology.

[CR65] Niu SJ, Niemi H, Harju V, Pehkonen L (2021). Finnish student teachers’ perceptions of their development of 21st-century competencies. Journal of Education for Teaching.

[CR66] Nousheen A, Kalsoom Q (2022). Education for sustainable development amidst COVID-19 pandemic: Role of sustainability pedagogies in developing students’ sustainability consciousness. International Journal of Sustainability in Higher Education.

[CR67] OECD (2022). OECD Better life index: Education. https://www.oecdbetterlifeindex.org/topics/education. Accessed 1 June 2022.

[CR68] Oommen PG (2020). Learning theories—Taking a critical look at current learning theories and the ideas proposed by their authors. Asian Journal of Research in Education and Social Sciences.

[CR69] Painter-Morland M, Sabet E, Molthan-Hill P, Goworek H, de Leeuw S (2016). Beyond the curriculum: Integrating sustainability into business schools. Journal of Business Ethics.

[CR70] Pokhrel S, Chhetri R (2021). A literature review on impact of COVID-19 pandemic on teaching and learning. Higher Education for the Future.

[CR71] Rashid L (2019). Entrepreneurship education and sustainable development goals: A literature review and a closer look at fragile states and technology-enabled approaches. Sustainability.

[CR72] Rodríguez Aboytes JG, Barth M (2020). Transformative learning in the field of sustainability: A systematic literature review (1999–2019). International Journal of Sustainability in Higher Education.

[CR73] Rof A, Bikfalvi A, Marquès P (2020). Digital transformation for business model innovation in higher education: Overcoming the tensions. Sustainability.

[CR74] Romestant F (2020). Sustainability agencing: The involvement of stakeholder networks in megaprojects. Industrial Marketing Management.

[CR75] Sachs J, Kroll C, Lafortune G, Fuller G, Woelm F (2021). Sustainable development report 2021.

[CR76] Sammalisto K, Sundström A, Holm T (2015). Implementation of sustainability in universities as perceived by faculty and staff—A model from Swedish university. Journal of Cleaner Production.

[CR77] Sandri O (2022). What do we mean by ‘pedagogy’ in sustainability education?. Teaching in Higher Education.

[CR78] Sangrà A, Wheeler S (2013). New informal ways of learning: or are we formalising the informal?. International Journal of Educational Technology in Higher Education.

[CR79] Saykili A (2019). Higher education in the digital age: The impact of digital connective technologies. Journal of Educational Technology and Online Learning.

[CR80] SDG (2015). The UN Sustainable Development Goals. https://www.undp.org/sustainable-development-goals. Accessed 27 June 2022.

[CR81] Seatter CS, Ceulemans K (2017). Teaching sustainability in higher education: Pedagogical styles that make a difference. Canadian Journal of Higher Education.

[CR82] Shin DH (2017). The role of affordance in the experience of virtual reality learning: Technological and affective affordances in virtual reality. Telematics and Informatics.

[CR83] Shrivastava, A. (2018). Using connectivism theory and technology for knowledge creation in cross-cultural communication. *Research in Learning Technology*, 2061.

[CR84] Siemens G (2005). Connectivism: A learning theory for the digital age. International Journal of Instructional Technology and Distance Learning.

[CR85] Siemens G, Hug T (2007). Connectivism: Creating a learning ecology in distributed environments. Didactics of microlearning. Concepts, discourses and examples.

[CR86] Simamora RM (2020). The Challenges of online learning during the COVID-19 pandemic: An essay analysis of performing arts education students. Studies in Learning and Teaching.

[CR87] Sivapalan S, Clifford MJ, Speight S (2016). Engineering education for sustainable development: Using online learning to support the new paradigms. Australasian Journal of Engineering Education.

[CR88] Skulmowski A, Rey GD (2020). COVID-19 as an accelerator for digitalization at a German university: Establishing hybrid campuses in times of crisis. Human Behavior and Emerging Technologies.

[CR89] Sterling S, Corcoran P, Wals A (2004). Higher education, sustainability and the role of systemic learning. Higher education and the challenge of sustainability: Contestation, critique, practice, and promise.

[CR90] Takala A, Korhonen-Yrjänheikki K (2019). A decade of Finnish engineering education for sustainable development. International Journal of Sustainability in Higher Education.

[CR91] Thota N (2015). Connectivism and the use of technology/media in collaborative teaching and learning. New Directions for Teaching and Learning.

[CR92] Tillmanns T (2020). Learning sustainability as an effect of disruption. Environmental Education Research.

[CR93] UNESCO (2022). Sustainable Development. https://en.unesco.org/themes/education-sustainable-development/what-is-esd/sd. Accessed 10 June 2022.

[CR94] Utecht J, Keller D (2019). Becoming relevant again: applying connectivism learning theory to today's classrooms. Critical Questions in Education.

[CR95] Verhagen, P. (2006). Connectivism: A new learning theory. https://jorivas.files.wordpress.com/2009/11/connectivismnewtheory.pdf. Accessed 21 June 2022.

[CR96] Verkasalo M, Daun A, Niit T (1994). Universal values in Estonia, Finland and Sweden. Ethnologia Europaea.

[CR97] Wals AE (2011). Learning our way to sustainability. Journal of Education for Sustainable Development.

[CR98] Waycott J, Bennett S, Kennedy G, Dalgarno B, Gray K (2010). Digital divides? Student and staff perceptions of information and communication technologies. Computers and Education.

[CR99] Webb A, McQuaid RW, Webster CWR (2021). Moving learning online and the COVID-19 pandemic: A university response. World Journal of Science, Technology and Sustainable Development.

[CR100] Zawacki-Richter O (2021). The current state and impact of Covid-19 on digital higher education in Germany. Human Behavior and Emerging Technologies.

